# Recent Advances in Scanning Electrochemical Microscopy for Biological Applications

**DOI:** 10.3390/ma11081389

**Published:** 2018-08-09

**Authors:** Luyao Huang, Ziyu Li, Yuntian Lou, Fahe Cao, Dawei Zhang, Xiaogang Li

**Affiliations:** 1 Institute for Advanced Materials and Technology, University of Science and Technology Beijing, Beijing 100083, China; hly_0531@163.com (L.H.); zyli16@163.com (Z.L.); louyuntian@hotmail.com (Y.L.); lixiaogang99@263.net (X.L.); 2 Department of Chemistry, Zhejiang University, Hangzhou 310027, China

**Keywords:** scanning electrochemical microscopy, biological system, living cells, biofilm

## Abstract

Scanning electrochemical microscopy (SECM) is a chemical microscopy technique with high spatial resolution for imaging sample topography and mapping specific chemical species in liquid environments. With the development of smaller, more sensitive ultramicroelectrodes (UMEs) and more precise computer-controlled measurements, SECM has been widely used to study biological systems over the past three decades. Recent methodological breakthroughs have popularized SECM as a tool for investigating molecular-level chemical reactions. The most common applications include monitoring and analyzing the biological processes associated with enzymatic activity and DNA, and the physiological activity of living cells and other microorganisms. The present article first introduces the basic principles of SECM, followed by an updated review of the applications of SECM in biological studies on enzymes, DNA, proteins, and living cells. Particularly, the potential of SECM for investigating bacterial and biofilm activities is discussed.

## 1. Introduction

Since it was first successfully established by Bard’s group in the 1980s [1], scanning electrochemical microscopy (SECM) has advanced considerably in various fields, especially for applications in biological systems. Although SECM originates from the concept of scanning tunneling microscopy (STM) [2], it differs from STM and other scanning probe techniques (e.g., atomic force microscopy (AFM) or scanning ion-conductance microscopy (SICM)). The key advantage of SECM is its ability to probe the diffusion layer on an electrode surface, which is achieved by an ultramicroelectrode (UME) with a diameter that is usually less than 25 μm [3,4]. The tip scans across an immersed substrate at a close distance, and the current response is recorded. Perturbations in the current response provide information on the nature and properties of the substrate. This capability makes SECM a powerful tool for quantifying the local concentration of different species on substrates and imaging the surface topography with a high resolution in liquid environments [5].

Since the first attempt to image the morphology of biological substrates [6], SECM has found wide applications in biological systems, including enzymes, DNA, proteins, and living microorganisms such as cells and bacteria. Over the past three decades, some excellent reviews have been published that provide an overview of the biological applications of SECM such as morphological imaging and monitoring specific chemical species in living cells [7,8,9,10,11,12,13,14,15]. In this review, following a discussion of the basic principles of SECM, we provide an update on the recent progress in tip fabrication techniques and instrumental development, and we discuss the combination of SECM with other scanning probe techniques, as in SICM-SECM and AFM-SECM. Next, the most recent progress in SECM is reviewed in detail in terms of its biological applications to the study of enzymes, DNA, proteins, and living cells. Particularly, the use of SECM to probe bacterial and biofilm activities is discussed.

## 2. Instrumentation and Operating Modes

**Figure 1:**
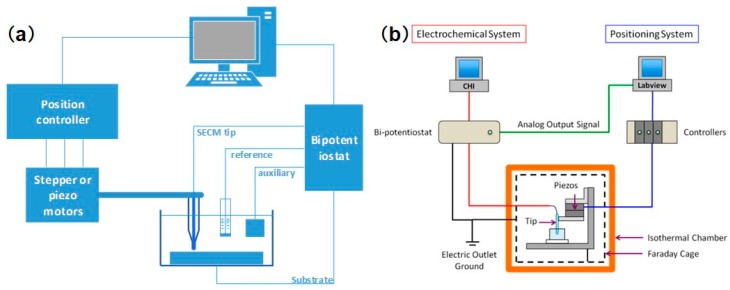
(**a**) Schematic diagram of the conventional scanning electrochemical microscopy (SECM) apparatus; (**b**) Schematic diagram of the modified SECM. Copyright (2016) American Chemical Society.

Generally, an SECM platform can be separated into several parts according to its functions. A schematic diagram is shown in Figure 1a. A low-current bipotentiostat provides precise potential and current control at the tip electrode and substrate. A three-dimensional (3D) positioning system consists of stepper and piezoelectric motors, which control the coarse and fine movement of the probe, respectively. The electrolytic cell used in SECM is usually homemade to satisfy specific experimental requirements. The probe controlled by the stepper and piezoelectric motors can move in the direction that is normal to the substrate to obtain a feedback curve, or it can scan across an immersed substrate to image the morphology of the substrate. A computer is used to operate the experiments and process the data. In addition to this basic setup, many studies have proposed combinations of SECM with a variety of complementary components, such as an inverted optical microscope [16,17], a temperature-controlling unit [18], an automatic motion control system [19], and an isothermal chamber [20,21], which are useful for studying the local electrochemical reactions and morphology variations in biological systems. However, with the development of nanometer-sized tips, this mature imaging technique is facing many new problems. The durability and precise control of tips have become obstacles to the further progress of this technology. To successfully implement the effect of nanometer-sized tips, Bard et al. [22] recently proposed an important advance in SECM instrumentation, as seen in Figure 1b. For the software design, an additional computer is assembled to operate custom-developed LabVIEW software, which controls the movement of the piezoelectric motor and synchronizes the tip motion with the tip current collected from the bipotentiostat. As for the hardware, all of the mechanical relay switches of the bipotentiostat were removed to avoid the transient amplification of high voltages, which may electrochemically damage the tip. Meanwhile, assembling lockable micropositioners along the x, y, and z axes may reduce undesirable tip drifting, thereby avoiding the distortion of the SECM image. The model they proposed, combined with the design of a nanoscale tip, provides us with an advanced approach to constructing nanoscale SECM equipment and investigating the sophisticated nanostructures of biological samples.

Since the initial attempts to image biological substrates, several operation modes, including the feedback mode, generation and collection mode, redox competition mode, potentiometric mode, and penetration mode have been proposed to investigate different samples, as discussed in the following section.

### 2.1. Feedback Mode

**Figure 2:**
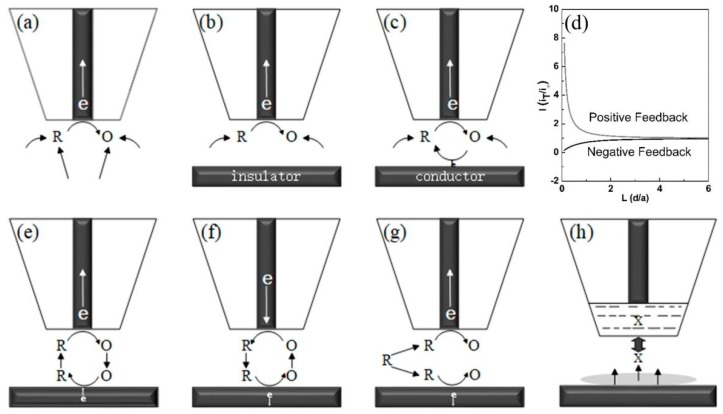
Different mode of the SECM operation. (**a**) Steady-state diffusion in bulk solution; (**b**) Negative feedback mode; (**c**) Positive feedback mode; (**d**) Approach curves of pure positive and negative curve; (**e**) Tip generation/substrate collection mode; (**f**) Substrate generation/tip collection mode; (**g**) Redox competition mode; (**h**) Potentiometric mode, where “X” is an ion in solution.

The amperometric feedback mode is the most fundamental operating mode among all of the modes that have been introduced since the invention of SECM. The realization of this mode depends on the characteristics of the UMEs and the substrate. As illustrated in Figure 2a, *R* represents a redox mediator in its reduced form, and an UME is immobilized sufficiently far from the substrate; i.e., normally, the tip-to-substrate distance (*d*) is at least 10 times longer than the probe radius (*a*). When imposing an appropriate potential on the UME, *R* in the solution is oxidized at the tip at a diffusion-controlled rate according to the following reaction:

(1) $$R-ne^{-}\to O,$$

The current measured at the tip, $i_{T,\infty }$, is given by Equation (2):

(2) $$i_{T,\;\infty }=4nFDc\alpha ,$$

where *n* is the number of electrons transferred in the reaction, *F* represents the Faraday constant, and *D* and *c* are the diffusion coefficient and the concentration of *R* in the solution, respectively.

The tip-to-substrate distance must always be close to a few tip diameters so that the hemispherical diffusion of *R* from the bulk solution to the tip is blocked by the target surface. As the tip is brought closer to an electrical insulator, the current measured ($i_{T}$) decreases monotonically because of the inhibition of radial diffusion (pure negative feedback, $i_{T}<i_{T,\;\infty }$; Figure 2b). In contrast, if the substrate is an electrical conductor, the *O* species formed in the tip diffuses towards the conductive substrate, and *R* is regenerated, resulting in an additional flux of *R* according to the following equation:

(3) $$O+ne^{-}\to R,$$

This process compensates for the loss of the blockage effect of the larger substrate and increases the current to be higher than the steady-state current (pure positive feedback, $i_{T}>i_{T,\infty }$; Figure 2c).

The two behaviors referred to above are based on a hypothetical condition wherein reaction (3) on the substrate is limited by the diffusion of oxygen from the tip to the substrate. The plot obtained by recording $i_{T}/i_{\infty }$ with a decreasing tip-to-substrate distance (*d/a*) is called the approach curve (Figure 2d). The bottom and top curves represent the pure negative feedback mode and the positive feedback mode, respectively. In biological applications of SECM, the feedback mode is frequently used to map the topography of the sample. In this case, the cytomembrane of cells is impermeable to specific mediators. When the tip scans across the cell protruding from the surface, a negative feedback current is obtained, which reflects the topographical information of the cells [23]. Conversely, positive feedback imaging can be used to identify specific protein and antibody spots on a microarray biosensor platform [24]. The feedback responses obtained from the modified spots are significantly different from the background area. Using this method, the charge-transfer property of different adenosine triphosphate (ATP)-treated peptide surfaces was recently monitored via SECM for the first time. After being decorated with unmodified ATP, Fc-CO-C6-ATP and Fc-CO-Lys-ATP, different feedback responses were recorded, which demonstrated the distinct electrochemical properties of the modified substrates [25].

### 2.2. Generation and Collection Mode

The generation and collection mode (GC mode) is another important and common SECM operating mode. This mode can be further classified as the substrate generation/tip collection (SG/TC) mode or the tip generation/substrate collection (TG/SC) mode, as shown in Figure 2e,f. In the SG/TC mode, the electrochemically active species are generated from the substrate and collected at the tip, which is polarized to an appropriate potential. In the simplest depiction of the SG/TC mode, currents are measured at both the tip and the substrate, and the tip and the substrate reactions can be described as the following equations:

(4) $$R-ne^{-}\to O,\;\mathrm{substrate}\;\mathrm{reaction}$$

(5) $$O+ne^{-}\to R,\;\mathrm{tip}\;\mathrm{reaction}$$

Usually, the size of the substrate is far greater than the size of the tip, and therefore results in a much larger diffusion layer near the substrate. In view of this aspect, the SG/TC mode is applicable to the detection of concentration profiles or chemical flux from a substrate. For biological applications, the SG/TC mode has been applied to investigate immobilized enzymes and map enzyme spot arrays, such as oxidoreductases and enzyme-labeled immunosorbent assays [26]. In contrast with the redox cycle of the feedback mode, the tip current in the SG/TC mode is primarily generated by the enzymatic reactions, which makes the SG/TC mode a sensitive way to quantify the enzymatic activity. However, the blockage caused by the geometry of the tip and the stirring of the moving tip to the diffusion layer may limit applications of the SG/TC mode for detecting enzymatic reactions [27].

This process is reversed in the TG/SC mode, wherein the substrate collects the species generated from the UME tip. Theoretically, the current collected at the substrate is close to zero immediately after applying bias potential at both the tip and the substrate. Thus, as can be seen in Figure 2e, the *R* generated at the tip diffuses to the substrate, and the current increases and reaches a steady state eventually. Once the steady state is achieved, compared with the SG/TC mode, the collection efficiency of the TG/SC mode is considerably higher. For the TG/SC mode, a modified version that is particularly useful for biological applications is the micropipette delivery–substrate collection (MD/SC) mode [28]. A micropipette is designed to be loaded with solution containing the species of interest. After specific species have been delivered to the target cells or biofilm by the micropipette, SECM can be used to monitor the response or quantify the uptake of the target biological system [29,30].

### 2.3. Redox Competition Mode

Since pioneering works by Schuhmann et al. [31], the redox competition mode (RC mode) has been extensively used to investigate biological processes. In this mode, the tip and substrate are polarized at a certain potential to compete for the same analyte in a relatively narrow gap, as shown in Figure 2g [32,33]. When scanning at a constant height in the vicinity of the substrate, the reductive current collected at the tip mostly remains constant above the inactive area. However, if the tip approaches the active area where an oxygen reduction reaction occurs, the current collected at the tip will decrease due to the consumption of dissolved oxygen by the substrates. The RC mode is extremely useful in the evaluation of oxygen reduction reactions on an oxidase-modified surface, which will be of value for the design of enzymatic biosensor or biofuel cells. Compared with the TG/SC and feedback modes, the RC mode shows higher sensitivity and better lateral resolution due to the reduced background current. Using this mode, Schuhmann et al. [34] monitored the local biocatalytic activity of bilirubin oxidase/Os-complex modified electrodeposition polymer spots. Recently, Ramanavicius et al. [35,36] proposed a mathematical model for the RC mode and successfully applied the RC mode to investigate glucose oxidase (GOx)-modified conducting and non-conducting substrates. However, when the tip is very close to the enzyme-modified conducting substrate, the regeneration of the measured redox species and the hindered diffusion of the redox species should be considered.

### 2.4. Potentiometric Mode

The above common operation modes are all based on amperometric methods, but in some specific circumstances, the potentiometric mode shows several obvious advantages for biological applications. In this mode, the measured signal is a potential, not a current. Thus, potentiometric measurements do not typically disturb or change the analytes under investigation, nor do they cause a mass transport variation of the analytes, which enables accurate measurements in biological systems with small volumes. In addition, the potentiometric mode can be used to monitor non-electroactive species in biological systems that are not accessible to amperometric methods such as hydrogen, alkali, and alkaline earth metal ions, which is a key advantage of ion-selective tips in addition to the high selectivity of their potentiometric signal.

In this mode, an ion-selective electrode (ISE) tip is typically used to detect the chemical systems of interest. Moreover, electrodes deposited with metal/metal oxide film are used in SECM for pH measurements, and serve as amperometric tips for distance control [37]. An ISE tip in an SECM measurement typically consists of an internal reference electrode and an ion-selective membrane (Figure 2h) [38]. When the ISE tip is placed into a sample solution containing the ion of interest, a concentration gradient of the analyte at the ion-selective membrane induces the formation of a junction potential, which can then be measured at the internal reference electrode. This specific chemical recognition of the analyte linearly depends on the activity of the analyte. Since no feedback effect forms in the potentiometric mode, to use potentiometric probes in SECM, an additional distance control system is needed to exactly control the tip-to-substrate distance. Initially, an optical microscope was used to monitor the tip position. Later, multi-barrel probes with separate amperometric and potentiometric channels were fabricated for simultaneous potentiometric scanning and distance calibration [39,40]. Recently, Nagy et al. [41,42] proposed new algorithms and deconvolution methods for high-speed scanning in potentiometric imaging, and discussed the relationship between scanning speed and image quality. These proposals represent an interesting attempt to image rapidly changing systems such as cross-membrane transportation and respiration activities, which is particularly useful for biological applications.

### 2.5. Alternating Current Mode

In contrast to the conventional amperometric SECM method, an alternating rather than constant potential is applied to the working electrode in alternating current mode (AC mode). To apply an alternating potential, a lock-in amplifier is assembled to produce a high-frequency sinusoidal wave, which is superimposed onto the constant potential provided by the potentiostat. The resulting AC response can be divided into two parts: the current magnitude I and the phase angle *θ*, which can be regarded as the real and imaginary parts of a vector, respectively. Analogously, negative and positive feedbacks also exist in the AC mode, wherein the AC current increases or decreases with the decreasing distance between the tip and the sample surface [43].

The AC mode provides several advantages for studies of biological systems. First, it does not need a redox species in the supporting electrolyte solution, which could prevent the toxic effect of a specific redox species on the investigated biological systems. In a conventional SECM study of living cells, accurate approach curves are not easy to obtain. The AC feedback mode can precisely calibrate the distance scale between the tip and the substrate [44]. The AC mode has been demonstrated to directly obtain topographical information from model neurons in a cell culture medium [45], and has been applied to monitor and identify GOx-based biosensor surfaces [46].

### 2.6. Penetration Mode

As new fabrication techniques emerge, nanoscale UMEs have become feasible, leading to a new SECM mode known as the penetration mode. As the name implies, a SECM tip can be used to penetrate an individual cell and measure the extracellular and intracellular electroactive species. The penetration mode provides a new way to explore intracellular activities such as ionic transfer and drug delivery, and may greatly enhance our understanding of cellular behaviors. Nanoscale probes are considered ideal SECM tips because of their ability to penetrate into living cells without destroying the normal cell functions. Actis et al. [47] reported a novel disk-shaped carbon UME, which can be used to penetrate a single cell and record electrochemical behavior with minimal disruption to the biological milieu. Using this nanoelectrode, they successfully monitored the oxygen concentration inside and outside a three-week-old brain slice. Similarly, Bau et al. [48] developed a carbon-based nanoelectrode and measured electrical signals in the mouse hippocampal cell line HT-22.

### 2.7. Probes

**Figure 3:**
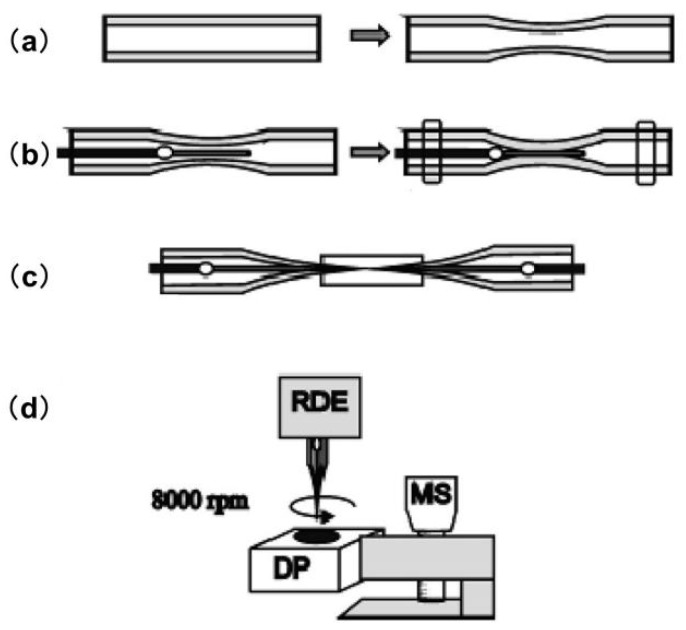
Protocol for microelectrode fabrication. (**a**) Quartz capillary thinning following laser exposure and pulling; (**b**) Insertion and sealing of a Pt wire in a quartz capillary; (**c**) Quartz capillary pulling process; (**d**) Mechanical micropolishing using rotating disk electrode (RDE), microsrew (MS), and diamond coated paper (DP). Copyright (2011) American Chemical Society.

The spatial resolution of SECM primarily depends on the size of the UME, which is commonly fabricated with carbon, platinum, gold, and other less-used materials such as silver and mercury. Usually, the SECM probes consist of an electroactive core surrounded by an insulating glass sheath. The ratio between the insulating sheath and electroactive core is defined as RG, which is an essential parameter for precise SECM measurements. To date, UMEs with diameters of 5–25 μm are commercially available, whereas finer UMEs can be obtained only by homemade methods such as electrochemical etching [49] or laser pulling techniques [50,51,52]. UMEs with sizes smaller than the diffusion layer ($\delta =\sqrt{\pi Dt}$, *D*: diffusion coefficient and *t*: time) present several significant advantages over macroscopic electrodes, including a reduced ohmic potential (iR) drop and a faster steady-state response [53]. A significant amount of SECM literature has focused on the design and fabrication of unique microscale and nanoscale UMEs that are capable of scanning in the vicinity of biological samples or even penetrating into cells [54,55,56]. Electrochemical etching is frequently used to fabricate UMEs from metals by readily reducing the size of the microwire to a few nanometers [57,58]. Another common strategy for fabricating nano-UMEs is laser pulling, which enables the simultaneous sharpening of the microwire and its surrounding sheath. Mauzeroll et al. published a reproducible procedure for obtaining UMEs with controllable sizes using a laser pulling technique [59]. Before the conventional sealing procedure, a quartz thinning step was added to enable the optimized elongation of the Pt wire, as shown in Figure 3. The Pt wire was then sealed inside the pretreated quartz capillary using a pipet puller, followed by a subsequent thinning step of the composite tube (platinum/quartz assembly). Finally, the composite tube was pulled to produce two UMEs with diameters ranging from 2 μm to 100 nm and an RG of less than 10.

Compared with metallic UMEs, carbon-based UMEs have shown several advantages for biological measurements, including their high current density, low ohmic drop, and minimal electrode fouling. Moreover, carbon electrodes possess much higher proton reduction overpotentials than most metallic electrodes, which means they can conduct electrochemical measurements over larger potential windows. Similar to most metallic UMEs, glass encapsulation was originally needed to seal the carbon fiber and fabricate disk-shaped carbon microelectrodes. The UMEs used in SECM should minimize the size and reduce RG as much as possible in order to closely approach the surface of the substrate. Therefore, different etching methods, including flaming and electrochemical approaches, were then proposed [60,61]. Pyrolytic carbon deposition, which is another fabrication strategy for carbon-based electrodes, exhibits excellent electrochemical behavior at various scan rates.

### 2.8. Multi-Sensing Platform

In a conventional SECM measurement, the signals collected from the tip depend on the topography variation and electrochemical reactivity of the substrate. To separate these two signals and independently monitor one of the two parameters, several advancements have been proposed to design SECM platforms with multi-sensing capacities.

A significant advance is the introduction of shear-force SECM (SF-SECM), which provides a new approach to controlling the distance between the substrate and the probe. Shear force-based positioning is derived from near-field scanning optical microscopy (NSOM), and was first introduced to SECM experiments by Schuhmann et al. [62]. In this mode, the tip was assembled with two piezoelectric plates that mechanically vibrated in the vicinity of the sample. By monitoring the variation in the amplitude of the tip vibration, a threshold value was set to regulate the distance between the tip and the sample surface. The authors later finished a series of works to simplify SF-SECM and improve its precision, and recently proposed a new four-dimensional (4D) SF-SECM mode [63,64,65]. This new mode allows the recording of multiple SECM images at a constant distance over a relatively short time and avoids tip crashing. Using this mode, the influence of the reaction rate at the tip on monitoring cell respiration activity was confirmed, and the real respiration activity of an individual HEK293 cell was imaged [66]. More recently, Sundaresan et al. [67] proposed a novel method to fabricate UMEs adopted in the SF-SECM mode. The yield of the fabrication process reached 60%, which was a significant increase from the prior yield of approximately 10%. Moreover, the higher yield, the average shear-force amplitude, and the sensitivity were also markedly improved from 0.5 (V/V) and 1.5 (V/V) to 1.25 (V/μm)/V and 3.16 (V/μm)/V, respectively, which greatly reduced the noise signals and made the SECM a more suitable tool for monitoring cell or tissue activity.

With the progress of high-resolution imaging using SECM, researchers began to focus on the fabrication of combined probes to simultaneously collect different signals, such as SICM-SECM and AFM-SECM. Kranz [68] summarized the recent progress in the design of these combined probes in a recent review. For biological research, these methods provide an advanced approach to quantitatively investigating physiological processes and measuring specific chemical species inside or on the surface of living cells. Some important recent examples in this field are reviewed in Section 3.1 and Section 3.2.

**Figure 4:**
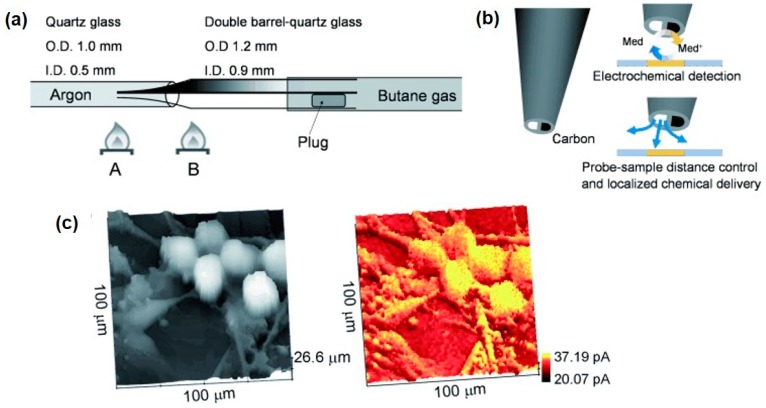
Double barrel carbon nanoprobes (DBCNP) fabricated by using the pyrolytic carbon deposition method. (**a**) Schematic illustration of the fabrication method of the DBCNP; (**b**) The principle of combined SECM-scanning ion-conductance microscopy (SICM) measurement with a DBCNP; (**c**) Simultaneous topographical (left) and electrochemical (right) images. Copyright (2011) John Wiley and Sons, Ltd.

SICM is a promising technique for noncontact topographical analyses that can be realized by injecting certain electrolytes into the glass/quartz nanopipette followed by insulation and exposure of the ring electrode at the end of the capillary [69]. SICM-SECM typically consists of double-barrel probes, of which one barrel is filled with electrolyte to control the SICM distance, and the other is a SECM carbon electrode measuring the uptake of the molecules of interest. The combined SICM-SECM imaging technique quickly received strong interest for studying dynamic biological processes because of its capacity for precise distance control and high spatial and temporal resolutions. However, several challenges remain in the preparation of proper SICM-SECM probes, including the issues of controlled geometry and reproducibility. Focused ion beam (FIB) milling was used to expose the probe with a reduced diameter of ~300 nm [70]. Recently, Takahashi et al. [71] presented a simplified fabrication method for a double-barrel SICM-SECM probe with an extremely quick fabrication time (<2 min) and a high success rate (Figure 4a). The effective radii of the two barrels were no more than 50 nm, and the overall radius of the probe was only 100 nm. The SICM channel scans over the target area for topographical information, and the SECM electrode records the electrochemical signal of the redox mediator, ferrocenemethanol (FcCH_2_OH). As shown in Figure 4c, the SICM and SECM images clearly presented the dendritic structures of living sensory neurons. In another report, the same group validated the capability of this novel probe for investigating the spatial distribution of neurotransmitter release together with the related variation in the cell topography that occurs during exocytosis [72].

**Figure 5:**
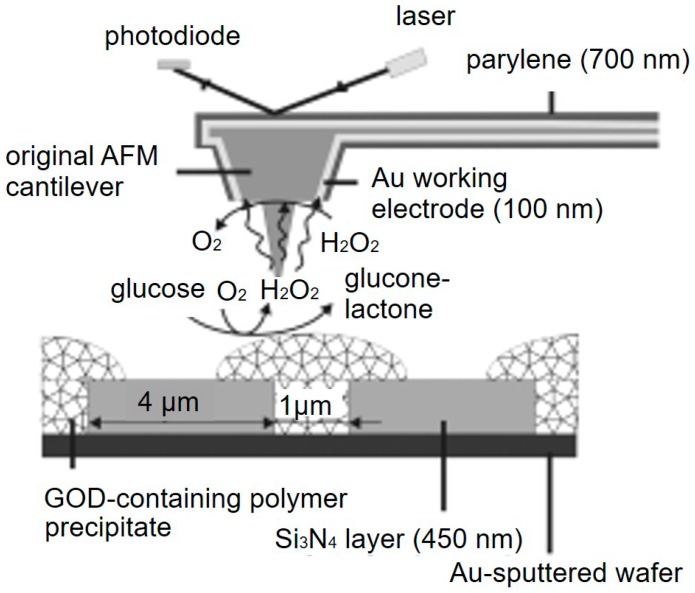
Schematic representation of simultaneous atomic force microscopy (AFM)-SECM imaging and the reactions involved at the surface of the micropatterned sample with the integrated electrode operating in generation–collection mode. Copyright (2003) John Wiley & Sons, Ltd.

AFM-SECM is another important SECM technique with potential biological applications. Compared with SICM, the combination with AFM not only creates excellent spatial resolution, it also provides synchronous nanoscale measurements of topographical changes and mechanical information on the specimen [73]. Several methods have been reported for fabricating AFM-SECM probes, including etching and insulating conductive microwires (Au or Pt) [74], plasma-enhanced chemical vapor deposition [75], and FIB milling [76]. The high spatial and topographical resolution of the AFM analyses coupled with the amperometric information obtained from an SECM section make AFM-SECM a reliable tool for studying complex live biological samples such as DNA chains and protein distributions [77]. Kueng et al. [78] investigated the enzymatic activity of immobilized GOx. As shown in Figure 5, the tapping mode of AFM and the generation-collection mode of SECM were applied to simultaneously record the variations in surface topography and electrochemical activity. In the presence of glucose, hydrogen peroxide was produced in the enzymatic conversion process, and an increased current was detected that corresponded to this reaction when the tip scanned above the enzyme spot. Kranz et al. [79] also imaged the enzymatic activity of immobilized horseradish peroxidase (HRP) using a homemade AFM-SECM probe. AFM-SECM was further applied to study the motional dynamics of single-stranded and double-stranded DNA by Demaille et al. [80]. Recently, they imaged the eIF4E factor, which is a component of the cellular translation initiation complex using AFM-SECM [81]. Moreover, a trifunctional SECM platform integrating the AFM-SECM-NSOM functionality has already been demonstrated and applied to imaging the neurites of living PC12 cells [82].

### 2.9. Modeling for SECM Modes

To better understand and interpret the experimental data collected from SECM, it is essential to develop theoretical models for different modes and experimental systems. For example, modeling is needed to confirm the influence of tip geometry on the SECM. In this case, the diffusion problem occurring in the gap between the tip and substrate is represented by a series of differential equations with initial and boundary conditions. More importantly, modeling approaches would improve SECM’s capability to provide more quantitative analyses on the local uptake rates or delivery rates of molecules of interest from biological substrates, as well as information regarding reaction kinetics, such as for example on enzyme-modified surfaces [35].

SECM has been proved to be an ideal electrochemical method, since it is not complicated to establish approximate diffusion equations describing the electron-flux between the tip–substrate gap or related issues with the electrochemical initial and boundary conditions [83]. Considering the complexity of the surface state of the biological substrates and the irregular geometry of novel tips for biological application, the application of modeling will become increasingly important to determine the experimental approach curves and further calibrate the normalized SECM currents. The models for different operation modes have been proposed using the finite element method (FEM) [84,85], finite difference method (FDM) [86], and boundary element method (BEM) [87,88].

With the progress in the tip fabrication technique, atypical SECM systems such as SICM-SECM and AFM-SECM have become prevalent. In these systems, the diffusion of the electroactive species toward the targeted electrode shows a clear distinction compared with conventional SECM platforms, resulting in a variation of the recorded currents in the targeted electrode. Rebuilding new models for these SECM systems is essential for quantitative interpretations of SECM results. This is especially true considering that these sophisticated SECM systems are normally homemade, and their tips do not often have regular geometry. Using FEM modeling, Unwin et al. constructed a two-dimensional (2D) model for a dual-channel nanopipette above *Zea mays* root hair cells with different initial and boundary conditions. They first simulated the SICM approach curves to a surface of zero uptake of redox mediator to set a 120-nm probe–substrate separation. Subsequently, simulations were performed with varying analyte uptake rates at the same height. Using the calibration curve obtained this way, the uptake of hexaammine ruthenium (III) to *Zea mays* root hair cells can be quantified, and different uptake rates can be obtained for the cell tip and cell body [30].

## 3. Applications

In biological sciences, not only imaging the topography of a sample topography, but also mapping specific chemical species, properties, and functions of the cell surfaces in time and spatially resolved manners is often desired. SECM is a noncontact, label-free tool that has impressively evolved from a low-resolution electrochemical imaging system after its first introduction by Bard et al. in 1989 to a high-resolution topographic imaging technique in recent years. Although SECM cannot compete with AFM or SEM in terms of topographical resolution, it is a powerful tool for differentiating the electrochemical activities of the different locations of the biological substrates on the sub-micron scale, as well as recently on the nanometer scale.

### 3.1. Imaging of Enzyme Activity

The first application of SECM to the study of enzymatic reactions was reported by Pierce et al., who measured the redox catalysis of GOx in 1992 [89]. Since then, different active enzymes, including diaphorase [90,91], HRP [92,93], glucose dehydrogenase [94], ceruloplasmin [95], and cytochrome c peroxidase [96] have been investigated via SECM with different operating modes, and enzymatic sensing has become a major application for SECM in the biological field.

During the past decade, enzymes have been widely used in enzyme-based biosensors, and some novel uses of SECM in designing enzyme-based biosensors have been proposed. Through various immobilization methods such as physisorption, cross-linking, and embedding in polymers, different enzymes have been immobilized on various substrates. Monitoring the catalytic activity of immobilized enzymes is very important for designing biosensors.

The activity of the immobilized cytochrome c was studied via SECM using [Fe(CN)_6_]^4*−*^ as the redox mediator [96]. The authors demonstrated that the maleimide–cysteine method offered better catalytic activity for cytochrome c than the conventional amide-linkage method, and they calculated the electron transfer kinetic parameters. Quantitative analysis of the surface-bound enzyme activity was performed by fitting the approach curves to the limited heterogeneous substrate kinetic model. Alternating current scanning electrochemical microscopy (AC-SECM) has also been applied to monitor and identify GOx-based biosensor surfaces [46]. In contrast with conventional SECM techniques, AC-SECM minimally disturbs the target analyte in the investigated solution. Due to the dependence of the AC signal at the tip of the tip-to-sample distance, AC-SECM can be applied to topographic imaging or for the positioning of the biosensor [97].

**Figure 6:**
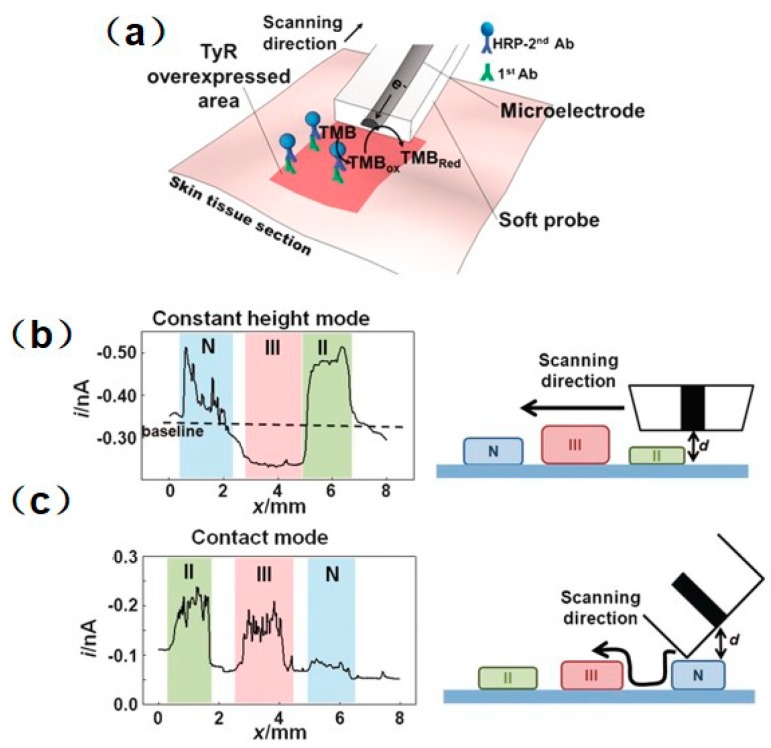
(**a**) Schematic representation of the immunoassay-based detection strategy to map the TyR distribution in tissue sections by using a soft SECM probe. SECM line scans in constant-height mode using a conventional microelectrode (**b**) and in contact mode with a soft SECM probe (**c**) over a tissue microarrays containing normal skin (N), stage II melanoma (II), and stage III melanoma (III) melanoma tissue sections. Copyright (2016) John Wiley & Sons, Ltd.

SECM is also used as a versatile platform to investigate enzyme activity associated with cancer or tumor cells, and as a useful complementary prognostic technique for distinguishing damaged and healthy cells. TyR is a vital enzyme that controls the production of melanin, and can be used as a highly selective indicator to monitor melanoma stages [98]. SECM was proposed for precisely mapping the TyR distribution in melanoma tissues [99]. To solve the swelling problem of the tissue and improve the measurement resolution in the use of a conventional Pt microelectrode, a soft-stylus probe was designed to conduct line scans over skin biopsy sections containing (1) normal; (2) melanoma stage II; and (3) melanoma stage III skin tissues. In contrast with a conventional microelectrode, the soft-stylus probe can scan along with the tissue sections without damaging the tissue from mechanical tip–tissue contact. Moreover, the contact scan mode can avoid the problem of reduced resolution in constant-height scan mode. As seen in Figure 6, the current signals collected above the pathological tissues were higher than those collected above the normal tissue, which indicates a higher expression level of TyR above the pathological tissues. In addition, a slightly lower expression of TyR was recorded above the stage III sample than above the stage III sample, which is considered to be the result of the homogeneous distribution of TyR in stage III melanoma. Similarly, a recent study measured the concentration of enzyme cofactors in a single cancer cell and a healthy cell [100].

**Figure 7:**
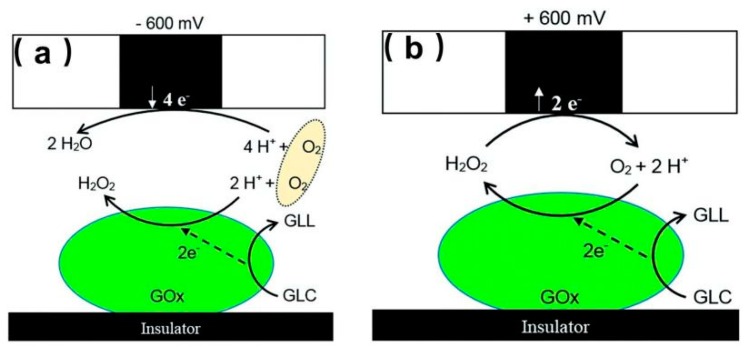
(**a**) Schematics of processes occurring during SECM measurements on both glucose oxidase (GOx)-modified and ultramicroelectrode (UME) surfaces in redox competition (RC)-SECM mode without any redox mediator; (**b**) Schematics of SECM processes occurring on GOx-modified and UME surfaces in generation and collection mode (GC) mode without any redox mediator. In the scheme, gluconolactone is abbreviated as GLL, and glucose is abbreviated as GLC. Copyright (2014) Royal Society of Chemistry.

The microbial fuel cell (MFC) is a new electrochemical system that exhibits great prospects over existing abiotic energy forms. However, the characterization of the bioelectrocatalytic activity of immobilized enzymes on specific surfaces is of great importance for designing the MFC [34,101]. GOx is an electroactive enzyme that could be used to modify electrodes and design enzymatic MFC; thus, identifying GOx on the electrodes is important. Recently, Dector et al. [102] designed a novel paper-based microfluidic blood fuel cell that was integrated inside a human immunodeficiency virus lateral flow assay. With the aid of SECM, they evaluated the enzymatic activity of the GOx electrode and proved the successful integration of this blood-based fuel cell. Ramanavicius et al. [103] investigated the activity of immobilized GOx on poly(methyl methacrylate)-based substrates using both RC and GC modes. Under RC-SECM, as shown in Figure 7a, a biased potential (−0.6 V versus Ag/AgCl) was applied at the UME to compete with the active spots of the substrate immobilized with GOx for dissolved O_2_. Despite the competing consumption of O_2_ on the UME and the active enzymatic spots, a positive +0.6 V (versus Ag/AgCl) potential was applied to the UME to catalyze the H_2_O_2_ oxidation reaction that occurs during a GOx-catalyzed reaction, as shown in Figure 7b. The results illustrate that the diffusion layer of the produced H_2_O_2_ (~600 μm) was thicker than that of O_2_ (~100 μm), which suggests that H_2_O_2_ diffused very far from the surface after the enzymatic reaction, whereas O_2_ was consumed close to the surface modified by GOx.

### 3.2. Detection of DNA or Hybridization

Compared with conventional optical methods such as fluorescence and chemiluminescence, SECM is a sensitive and low-cost electrochemical tool for tracking and measuring DNA or RNA. Since the first attempt to visualize DNA microarrays [104], different groups have successfully applied SECM to investigate DNA behavior, including single-stranded DNA (ssDNA) [105,106], double-stranded DNA (dsDNA) [107,108], and DNA hybridization [109,110].

**Figure 8:**
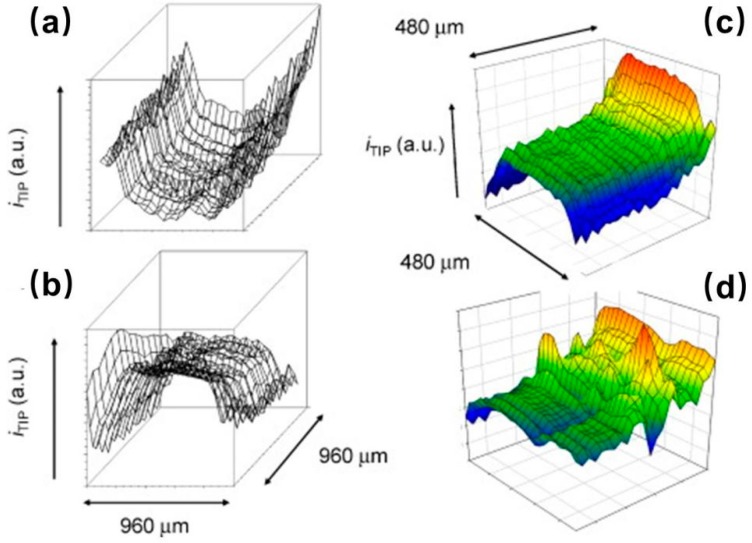
SECM images of (**a**–**c**) single-stranded DNA (ssDNA) and (**d**) Double-stranded DNA (dsDNA) fibers immobilized on Pt substrate electrode in contact with a 2-mM ferrocene plus 1-mM L solution in 0.10 M Bu_4_NPF_6_/DMSO. (**a**) E_S_ = +0.65 V; E_T_ = +0.40 V; (**b**–**d**) E_S_ = −1.25 V, E_T_ = −0.45 V. Copyright (2014) Elsevier Ltd.

High-throughput DNA analysis using SECM can be achieved by separating the DNA signal supporter and inceptor with the aid of a four-electrode system. Meanwhile, the introduction of SECM considerably eliminates the disturbance of a double-layer charging current, which suppresses the electrochemical signal of the redox probe [111]. Accurately identifying and discriminating between ssDNA and dsDNA is important for evaluating DNA damage [112] and denaturing/renaturing processes [113]. Doménech-Carbó et al. [114] proposed a new application of SECM to distinguish dsDNA and ssDNA using canthin-6-one and ferrocene as redox mediators. As shown in Figure 8, the tip current of the boundaries of the ssDNA fibers was higher than those of the center, indicating a lower oxidation efficiency of ferrocene at the central ssDNA fibers. However, when a relatively lower potential was applied to the tip to record the reoxidation current of canthin-6-one that was reduced in the substrate, a higher current peak was obtained in the central region. This may be indirect evidence of a strong affinity between canthin-6-one and ssDNA. Some distinctions occurred when ssDNA and dsDNA fibers were immobilized on the substrate. The reoxidation current of reduced canthin-6-one above the dsDNA fibers showed intense fluctuations, which may be attributed to stronger binding between dsDNA and canthin-6-one than between canthin-6-one and ssDNA.

**Figure 9:**
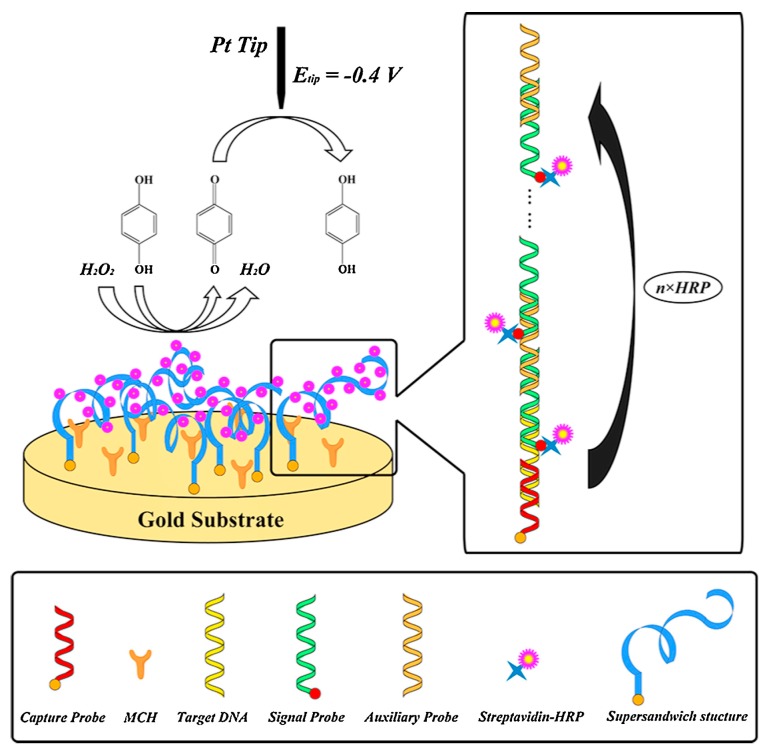
Schematic of the fabrication and SECM detection of the proposed DNA biosensing platform. Copyright (2016) Elsevier Ltd.

For more than a decade, researchers have tried to establish more sensitive and miniaturized SECM platforms for the detection and measurement of DNA microarrays, for which different signal amplification strategies have been developed. Using ferrocyanide as an SECM mediator and methylene blue as a redox-active intercalator, Zhou and Wain [115] increased the tip current for the detection of DNA microarray spots. Streptavidin–HRP, which is an efficient catalyst to oxidize hydroquinone (H_2_Q) to benzoquinone (BQ), was linked to the long DNA concatemers through the streptavidin–biotin interaction to amplify the SECM signal [116]. Recently, based on this HRP catalytic reaction, an ultrasensitive SECM platform for DNA biosensing was fabricated through a sequential self-assembly of DNA concatemers (Figure 9) [111]. No current signal was found at the initial step with only a capture probe (CP) assembled. Meanwhile, the current signal was evidently amplified in the presence of target DNA (TD) with the addition of a signal probe (SP) and an auxiliary probe (AP), which was attributed to the linkage of HRP to the DNA concatemers. Using a traditional 25-μm Pt UME, obvious current peaks were detected when scanning across the confined spots of a DNA microarray where specific hybridization occurred. The method proposed by the authors shows great potential for improving SECM sensitivity for high-throughput DNA detection, which is of great interest in drug design [117] and the diagnosis of genetic diseases [118].

SECM has also been used to detect single base mismatches (SBMs) in sandwiched dsDNA, which are the most frequent genetic variation within the human genome [119]. For example, Kraatz et al. [120] explored the probable positions of SBMs in dsDNA films by analyzing the electrochemical response of SECM. According to the electrochemical response of the apparent rate constant, they divided these positions into two broad groups, which were located in positions near spots five and nine and near spots one, 13, and 23. More recently, Mousavi et al. [121] reported an improved application of SECM for the specific detection of SBMs. Through the covalent attachment of ferrocenecarboxylic acid on the top of dsDNA, an electrocatalytic redox cycle formed at the gap between the SECM tip and the DNA-modified electrode, which was sensitive to the integrity of the DNA double helix. The presence of SBMs in the dsDNA double helix interrupted the regeneration of ferricyanide and thereby decreased the electron transfer rate, which could be captured by the SECM tip. Based on this method, an apparent standard electron transfer rate constant of different SBMs can be obtained. This specific detection based on the DNA-mediated electron transfer concept makes SECM a promising tool for detecting and differentiating different SBMs.

### 3.3. Investigation of Protein Activity

Compared with the growing commercial applications of DNA-based SECM testing platforms, the study of proteins via SECM remains in its infancy. This is in part because of the intrinsic instability and mutability of proteins when being immobilized onto substrates [122,123]. For more than a decade, different SECM-based analytical testing platforms have been developed for the study of proteins and their building blocks, including amino acids [124,125], cytochrome [126,127], antibody and antigen [128], leukocidin [129], peptides [130,131], and bacterial flagellin [132]. In addition to the detection of a single protein type, the determination of multiple proteins using SECM was recently reported. For example, Matsue et al. simultaneously detected two different proteins, pepsinogens 1 and 2, which are associated with atrophic gastritis and gastric cancer, respectively [133]. In another study, four tumor markers in lung cancer, namely, alpha-fetoprotein, carcinoembryonic antigen, neuron-specific enolase, and cytokeratin-19-fragment, were simultaneously detected for the first time using the GC mode of SECM [134].

**Figure 10:**
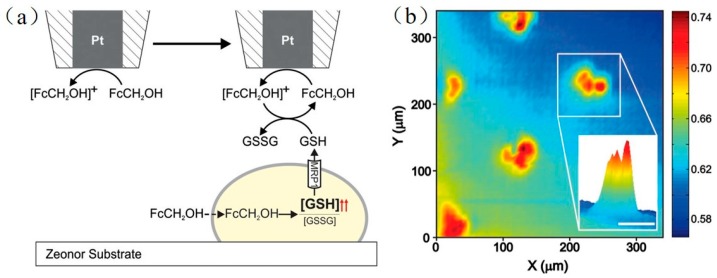
(**a**) Schematic illustration of constant-height feedback mode SECM imaging with a living cell in the presence of FcCH_2_OH as a redox mediator; (**b**) Normalized SECM current recorded at a distance of 12 μm above the substrate when HeLa cells were exposed to FcCH_2_OH (1 mM) for 75 min. (Inset) Close-up three-dimensional (3D) view of the signal obtained from an individual cell island. Copyright (2013) National Academy of Sciences.

For almost every type of cancer cell, multidrug resistance is a common defense mechanism. Through non-selective transmembrane proteins, which serve as molecular “pumps”, chemotherapy drugs are extruded from cancer cells. To probe multidrug resistance mechanisms, SECM was used in the feedback mode to track the multidrug resistance-related protein 1 (MRP1) in patterned adenocarcinoma cervical cancer cells [135]. To ensure the uniformity of the patterned cancer cells, they designed a cell co-culture model to form islands of cancer cells in a side-by-side format, and calculated the analytical approximation for the negative feedback current. In the study, the authors used FcCH_2_OH as the redox mediator. FcCH_2_OH is cell-permeable, and can be bound specifically with glutathione (GSH), which is a peptide molecule involved in MRP1 protein-related transport. The unique interaction between FcCH_2_OH and glutathione (GSH) was utilized to quantitatively detect the activity of MRP1. As shown in Figure 10, when the tip scans across the patterned cells, the FcCH_2_OH inside the cells accelerate the overproduction of GSH, which is then expelled from the cells by MRP1. Exiled GSH joins in the FcCH_2_OH/[FcCH_2_OH]^+^ redox cycle, and finally increases the electrochemical signal.

**Figure 11:**
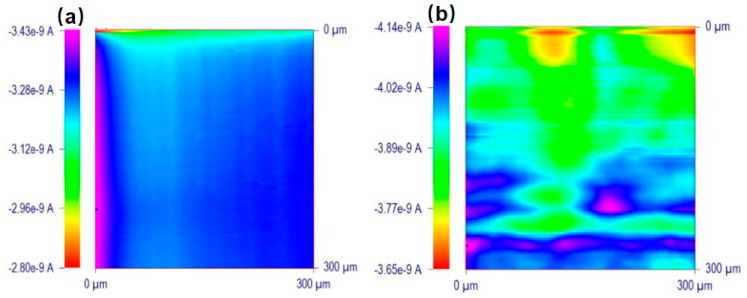
SECM 2D images obtained with a platinum microelectrode (a = 5 μm) in 1 mmol L^−1^ of Fe(CN)_6_^3−/4−^ in the presence 10^−1^ mol L^−^^1^ of KCl for a substrate consisting of 11-mercaptoundecanoic acid (1 mmol L^−^^1^) (**a**) and consisting of antigen (50 μg mL^−^^1^) immobilized on self-assembled monolayers (**b**). Copyright (2013) Elsevier Ltd.

SECM is also applied to probe the interaction between antigens and their antibodies, which was first reported in Higson’s work on the label-free imaging of arrays of immobilized biotinylated antibodies and detecting the binding of the neuron-specific enolase (NSE) antigen [136]. To fabricate the polyethyleneimine (PEI) arrays on the carbon electrodes, a homemade glass capillary filled with 1% biotinylated PEI solution was used. In situ imaging of the well-organized arrays before and after exposure to the NSE showed obvious differences, indicating the specific binding of the antigen to the antibody-derivatized dots. Through this successful use of SECM in detecting the interaction between NSE and biotinylated antibody, the authors proposed a new application of SECM to fabricate miniaturized antibody-based analytical testing platforms. Instead of detecting antigens, Damos et al. [137] recently developed an immunosensor to detect an anti-*Leishmania infantum* antibody using SECM. First, an 11-mercaptoundecanoic acid self-assembled monolayer was constructed on a bare gold electrode, followed by the immobilization of soluble *L. infantum* antigens in the SPR system. In the presence of ${\left[\mathrm{Fe}{\left(\mathrm{CN}\right)}_{6}\right]}^{4-}$, the authors imaged the modified gold disks with and without the antigen (Figure 11). A relatively fluctuating current change was observed, indicating that specific binding existed between anti-*L. infantum* antibodies and antigens. Therefore, the authors concluded that their methodology was promising for sensing visceral leishmaniasis in endemic regions.

### 3.4. Living Cell Studies

A wide variety of analytical methods have been developed to investigate biological processes in living cells in recent years, but challenges in living cell analysis remain because of the complexity of the intracellular or intercellular dynamic processes [138]. Depending on the non-invasive detection modes and high-resolution imaging capability, SECM has been used extensively to study living cells such as human cells [139,140,141,142] and animal cells from rats [143], dogs [144], and cows [145]. Using the approach curve enables accurately positioning the probe above the investigated cells and obtaining kinetic information on the redox species across the cell membrane [146]. As the size of the tips decreases, single cell measurements and even intracellular investigation become possible. Different redox mediators, including FcCH_2_OH [147], menadione/menadiol [148], and oxygen [147], have been shown to possess the ability to penetrate into the cell membrane, and can therefore be used to measure the intracellular enzymatic activity. Through 2D or three-dimensional (3D) cell imaging, the cellular topography and the spatial profile of extracellular redox-active species can be obtained [149,150].

**Figure 12:**
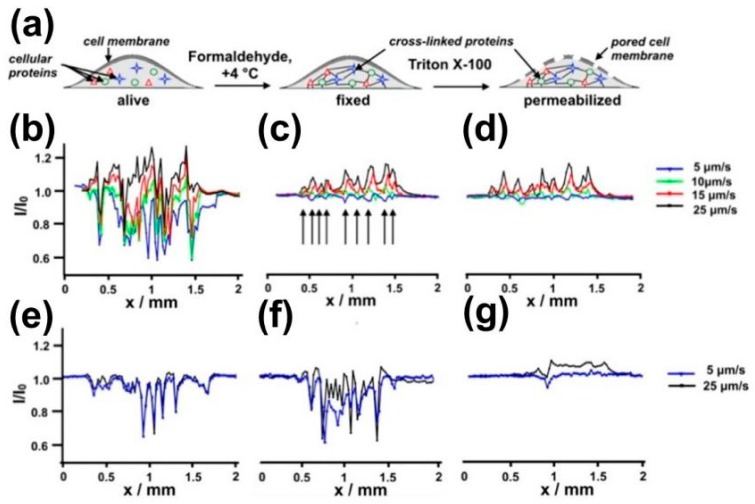
(**a**) Schematic representation of alive, fixed, and permeabilized cell. Influence of the UME translation speed on the SECM response (normalized current) provided by alive (**b**,**e**), fixed (**c**,**f**), and permeabilized (**d**,**g**) adherent WM-115 melanoma cells in the presence of noncharged (FcCH_2_OH, (**b**–**d**)) and charged (FcCOOH, (**e**–**g**)) redox mediators. Copyright (2016) American Chemical Society.

SECM was recently used to visualize the targeted intracellular biomarkers on fixed and permeabilized cells [151]. The passive transport of the redox mediator into the untreated, fixed, and permeabilized cells was investigated using SECM line scan tests above the cells (Figure 12), and the influence of different UME translational rates on electrochemical signals of the same cells was discussed. Using hydrophobic FcCH_2_OH as a redox mediator, identical amplified current profiles were obtained when applying an incremental speed from 5 μm/s to 25 μm/s on fixed and permeabilized cells. However, when the hydrophilic redox mediator FcCOOH was used, the current profiles of fixed and permeabilized cells showed opposite changes. This finding was partly due to the forced convection introduced by the fast UME movement and the intrinsic biological activity of the investigated cells.

**Figure 13:**
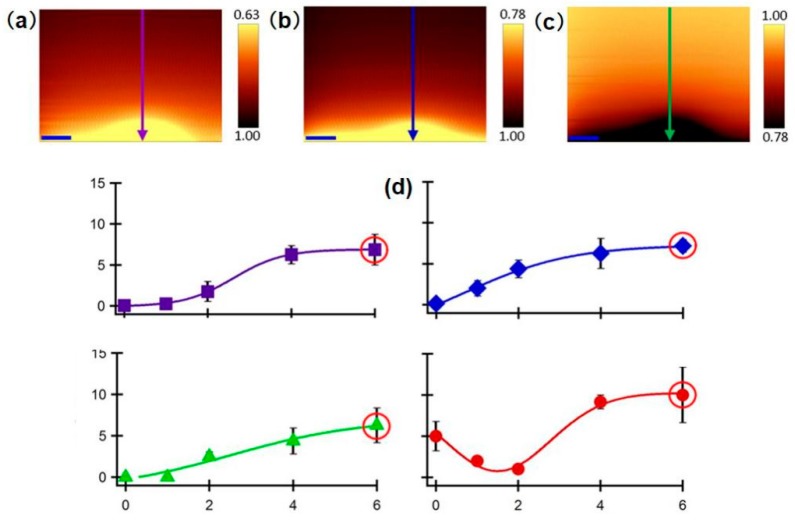
Typical SECM depth scan images of single T24 collected using redox mediators: (**a**) FcCOO^−^, (**b**) Fc(COO)2^2*−*^, and (**c**) Ru(NH3)6^3+^. (**d**) Average membrane permeability of T24 cells preincubated with 25 μM of Cd^2+^ as a function of incubation time using the following molecular probes: FcCOO^−^ (purple squares), Fc(COO)_2_^2*−*^ (blue diamonds), Ru(NH3)_6_^3+^ (green triangles), and FcCH_2_OH (red circles). Copyright (2016) American Chemical Society.

Heavy metal cytotoxicity has recently been investigated using SECM. For example, Ding et al. [152] investigated the influence of Cd^2+^ concentration and immersion time on the membrane permeability of human bladder cancer (T24) cells. Hydrophilic FcCOO^−^, Fc(COO)_2_^2*−*^, and Ru(NH_3_)_6_^3+^ were used as redox mediators, and the approach curves in the vertical direction showed that they were impermeable to the cells. Then, the authors further measured the membrane permeability of T24 cells after 0 h to 6 h of incubation with 25 μM Cd^2+^. The membrane permeability to these hydrophilic redox mediators increased and maintained a similar maximum average permeability coefficient after 6 h of incubation, as shown in Figure 13. With sufficient Cd^2+^ treatment, the T24 cell membrane integrity weakened, and within an hour, the membrane permeability to these redox mediators reached approximately 7.0 × 10^−^^5^ m/s, or 2.0 × 10^−^^4^ m/s in severe cases. The qualitative current results of membrane permeability measurement are not enough to provide detailed information of the effects of toxin. To quantify the membrane permeability coefficients of these hydrophilic redox mediators under the treatment of Cd^2+^, the authors established a simulation model in 2D axial symmetry using COMSOL Multiphysics. The model geometry, including the tip size and the shape of the electrolyte cell, were redesigned to be representative of the physical dimensions of all of the elements in this biological SECM system [153,154]. In this model, the approach curves showed less negative feedback characteristics with an increasing permeability coefficient from 0 to 1.0 × 10^−^^3^ m/s. Using this normalized approach curve, the experimental approach curves can be extracted, and the changes in electrochemical behavior can be transferred to quantitative permeability coefficients.

**Figure 14:**
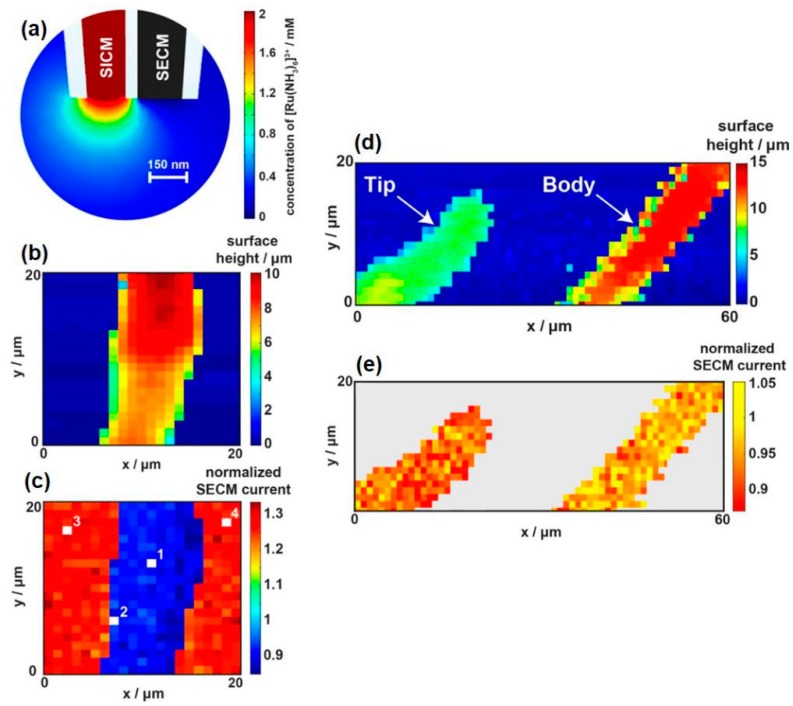
(**a**) Finite element method modeling of the SICM-SECM uptake system; (**b**) Substrate topography extracted from the z-position at the point of closest approach; (**c**) Normalized SECM current map showing the difference in uptake between glass substrate (zero uptake) and the root hair cell; (**d**) Substrate topography extracted from the z-position at the point of closest approach from the SICM channel; (**e**) Normalized SECM current map showing a clear difference in uptake between the root hair cell body (higher current, lower uptake) and the root hair cell tip (lower current, higher uptake). Copyright (2017) American Chemical Society.

Dual-channel nanoprobes have emerged in the last few years and have exhibited several advantages in living cell studies, such as relatively independent subsidiary working systems and improved sensitivity. In a typical example, one barrel is filled with electrolyte to control the distance, and the other barrel is an SECM electrode measuring the uptake of molecules of interest. Page et al. [89] fabricated the dual-channel SICM-SECM nanoprobes and simultaneously measured the topography and molecular uptake of Zea mays root hair cells (shown in Figure 14). In their system, one barrel was filled with electrolyte and molecules of interest for mapping topographical variation and delivering given molecules (SICM channel), whereas the other barrel was a solid carbon electrode measuring the uptake of molecules of interest (SECM channel). Using the feedback mode, they measured the topography of the selected rectangular region through the SICM channel and calculated the size of the cell. Meanwhile, a normalized SECM current map was obtained. When scanning above a living cell, a lower normalized current was obtained due to the uptake of Ru(NH_3_)_6_^3+^ across the membrane. As shown in Figure 14, this accurate mapping system can even distinguish the different regions of the cell. The root hair cell was bent in a hairpin shape to satisfy the simultaneous imaging of the root hair tip and body. The normalized SECM current value of the labeled body was higher than that of the labeled tip. Cell membrane permeability or membrane transport proteins may explain the different rates of uptake. The authors proposed a new idea and concluded that their methodology was useful for screening drug molecules, which is essential to understanding drug efficacy.

### 3.5. Study of Bacterial Behavior and Biofilm Activity

Compared with living cells, the SECM study of single living bacterial cells remains challenging because of their much smaller cell sizes. For bacterial studies, multi-cell measurements are often used to investigate bacterial substrates because of the difficulty of precisely positioning nanoscale probes over the small-diameter bacteria. Microbial cells that grow on the surfaces often aggregate in a hydrated polymeric matrix that they synthesize to form a biofilm; such cells are physiologically distinct from those living in the planktonic state [155,156]. SECM has the unique ability to measure different redox species, such as some active metabolites in the biofilm, at an exact distance over the investigated biofilm. In the SECM application to biofilms, the controllable formation of biofilms is a key factor, especially for biofilms grown on a metal surface. How to form a nearly natural, uniform biofilm on a metal electrode remains a significant challenge. Unlike SECM observations of biofilm grown on agar-containing nutrients, microorganisms exist in relatively low density on a metal surface. As a result, the concentration of signal molecules is often low, and can be difficult to detect and easily disturbed by the background signals from metal substrates and electrolytes [157,158,159]. Wessel et al. summarized the technologies that are available for characterizing the local chemical environment surrounding bacterial communities and highlighted the unique capability of SECM in quantitatively monitoring the redox-active molecules in biofilms [160]. Over the past several years, several different bacteria have been investigated, including pathogenic bacteria such as Escherichia coli [161,162], Staphylococcus aureus [163], Salmonella typhimurium [164]*,* and environmental bacteria such as Rhodobacter sphaeroides [165] and Paracoccus denitrificans [27].

**Figure 15:**
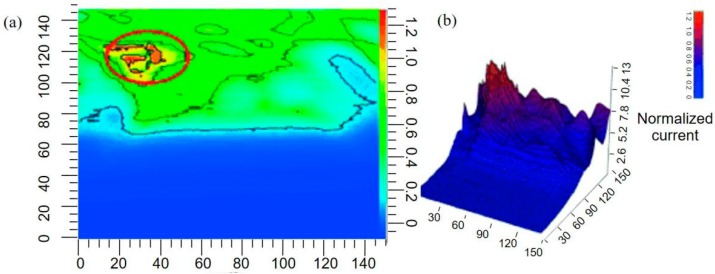
Real-time areascan series of a bottom gold electrode after 37 hours of bacteria electrogenic experiments. (**a**) The contour map, whereas (**b**) is the corresponding 3D image. Copyright (2015) John Wiley and Sons, Ltd.

SECM has been applied to monitor the biofilm formation and development on different substrates. This dynamic process could not be tracked or recorded by traditional electrochemical or imaging methods such as SEM and fluorescence microscope. For example, the formation of biofilms on MFC electrodes and their geometric status are vital factors for the electrogenesis efficiency. Zhang et al. [166] used SECM to study the formation process of Shewanella biofilm on an electrode during bacterial electrogenesis (Figure 15). As the bioelectrogenic process continued, the tip moved farther in the vertical direction to trigger positive feedback. Meanwhile, the feedback current decreased at each vertical point. The current change was determined by two factors: the increased electrochemical activity and the shielding effect of the insulating biofilm, which increased and decreased the current, respectively. This result proved that SECM characterization can accurately reflect the thickness and conducting properties of the biofilm.

Recently, Koley et al. [167] proposed an interesting application of SECM involving dual-channel electrodes with pH and Ca^2+^ sensors to monitor the local chemical process of urea hydrolysis and the subsequent CaCO_3_ precipitation by the living biofilm of Sporosarcina pasteurii. Compared with the pH of the bulk solution, the average pH in the vicinity of biofilm was 8.9, which decreased by nearly 1 after 30 min of exposure to urea at a different concentration. This result corresponded well to the Ca^2+^ vertical scan profile, indicating the occurrence of pH-induced CaCO_3_ precipitation. These recent advances provide an opportunity to design empirical models for specific bioremediation processes on biofilms.

**Figure 16:**
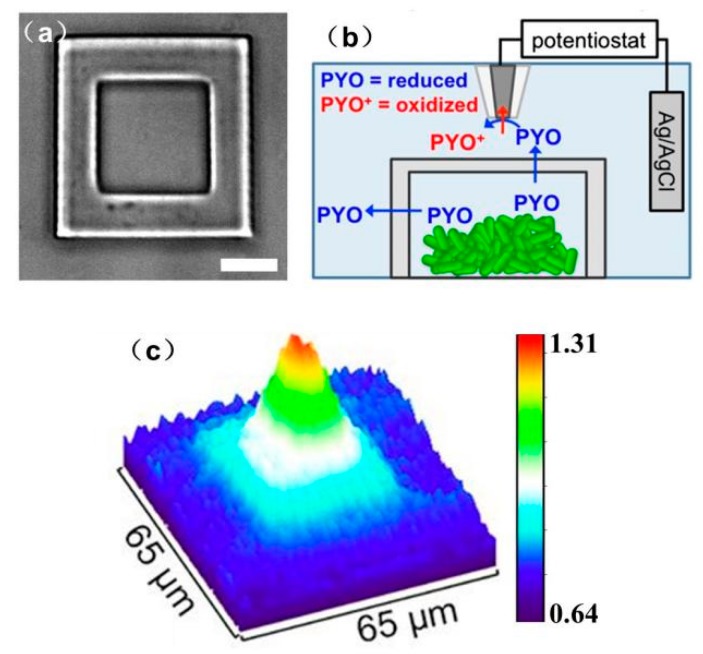
(**a**) Bright-field image of an empty 3D-printed microtrap. The trap as an 8-pL inner chamber (20 μm × 20 μm × 20 μm; length × width × height) that is surrounded by four 8-μm thick walls and a 3-μm thick roof. (Scale bar: 10 μm) (**b**) Schematic of the microtrap-SECM system for measuring PYO in real time. (**c**) A representative SECM image for pyocyanin (PYO) collected above a microtrap containing WT P. aeruginosa. Copyright (2014) National Academy of Sciences.

Quorum sensing (QS) communication between bacterial aggregates is a system of stimuli and response correlated to population density. As the bacteria population increases to a certain density, QS communication is triggered, which enables bacteria to coordinate the expression of specific genes and regulate aggregate growth [168]. The mechanism underlying QS communication has received intensive research interest in recent years. SECM can capture the information of subtle variations in small-molecule production by bacterial biofilms or record instantaneous perturbation, making it a powerful tool for establishing a relationship between the bacterial population density and the QS signals and investigating how bacterial spatial organization influences the interactions within microbial populations at the molecular level [169]. To determine the minimum bacterial population that can trigger QS communication, Whiteley et al. [170] investigated the QS of Pseudomonas aeruginosa placed in a microscale 3D-printed cage. As shown in Figure 16, a 5-μm diameter UME was positioned 2 μm above the roof of the cage containing a varying number of bacterial aggregates. Pyocyanin (PYO), a quorum sensing metabolite, was used as a proxy for QS-mediator communication. The release of PYO was measured at the SECM tip as the population grew over time. The current response in the tip confirmed that a minimum number of 500 bacterial cells was required for P. aeruginosa to initiate QS. In addition to measuring PYO production by bacterial aggregates, they also investigated the QS communication process between adjacent bacterial aggregates. In contrast with an individual aggregate, at least 2000 cells were needed to stimulate QS between adjacent P. aeruginosa aggregates.

**Figure 17:**
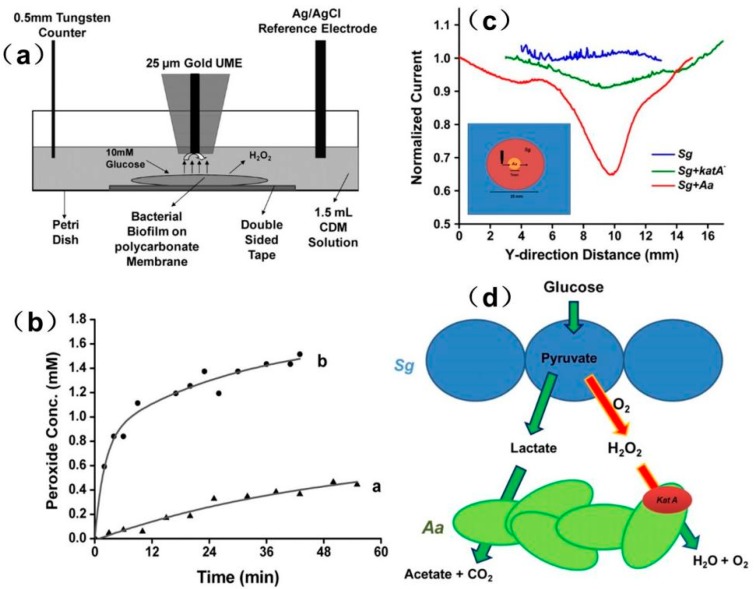
(**a**) Schematic diagram of an electrochemical experimental setup for the real-time measurement of hydrogen peroxide formation in a bacterial biofilm; (**b**) Plots of the concentrations of hydrogen peroxide produced by Streptococcus gordonii (Sg) in biofilm as a function of time at different distances between the Au UME and the biofilm (**a**, 200 μm, **b**, 100 μm); (**c**) Normalized current changes of an SECM y-scan over Sg alone, mutant Aggregatibacter actinomycetemcomitans (Aa) in an Sg film, and wild-type Aa in an Sg film, respectively. Inset is the schematic diagram of a tip scan across the region of a mixed species biofilm in the order of Sg–Aa–Sg; (**d**) Model for the role of hydrogen peroxide in an Sg and Aa co-cultured biofilm. Copyright (2011) National Academy of Sciences.

In a recent study, the local H_2_O_2_ concentration produced by Streptococcus gordonii (Sg) biofilms was measured in real time using a homemade SECM [171]. As shown in Figure 17, an apparent difference in the H_2_O_2_ level was detected at different positions away from the biofilm. With the help of FEM modeling, the exact H_2_O_2_ flux at the biofilm surface was quantified to be approximately 1.2 × 10^−^^19^ mol/s from a single bacterium at the film surface. The authors also studied the interaction between Sg and Aggregatibacter actinomycetemcomitans (Aa) in co-cultured conditions. Using line-scan experiments, the current response over different regions across the co-cultured biofilm was imaged. The y-direction scan revealed a different concentration profile across different regions of co-cultured biofilm. The sharp reduction in the current over the co-culture environment was due to the decomposition of Sg-produced hydrogen peroxide by Aa using the KatA enzyme, which can be explained by the model described in Figure 17. When the probe moved over AaKatA-, no significant decrease in the current was observed, thereby indicating that hydrogen peroxide was not consumed in the absence of Aa.

**Figure 18:**
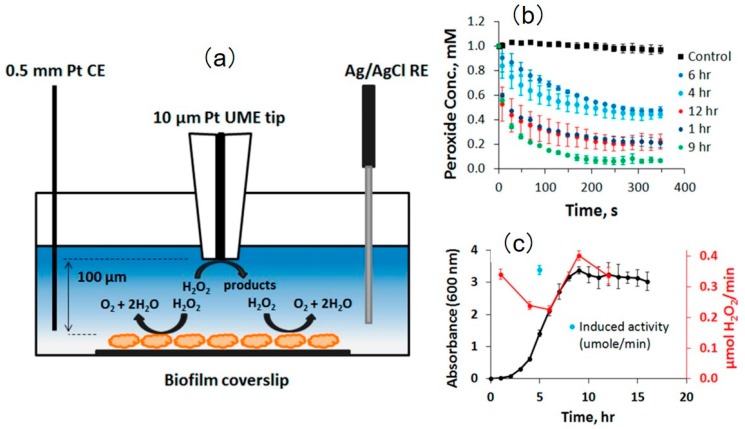
(**a**) Schematic of the electrochemical experimental setup for the SECM real-time measurement of hydrogen peroxide decomposition by a bacterial biofilm; (**b**) Hydrogen peroxide decomposition activity on *V. fischeri* CB37 biofilms at different incubation times; (**c**) Comparison of growth curve (**left** axis, black line, and solid dots) for planktonic *V. fischeri* CB37 bacteria and hydrogen peroxide decomposition activity (**right** axis, red line, and solid dots) on *V. fischeri* CB37 biofilms at designated incubation times. Copyright (2013) American Chemical Society.

The decomposition of H_2_O_2_ over Vibrio fischeri biofilm was also monitored using SECM. Zoski et al. [172] investigated the relationship between the external H_2_O_2_ concentrations and the catalase activity of the biofilm. Using O_2_ as a mediator, the approach curve was obtained, and the tip was then maintained at a height of 100 μm above the biofilms (Figure 18). Then, 1 mM of hydrogen peroxide was added to the bulk solutions incubated with *V. fischeri* CB37 and *V. fischeri* ETBB10-1. The decomposition process of H_2_O_2_ by both biofilms was recorded. In the presence of biofilms, the concentration of H_2_O_2_ decreased with time at different incubation stages compared with the control groups. However, the authors recorded a nonlinear variation in the concentration of H_2_O_2_ above *V. fischeri* CB37 biofilm with incubation time, which was caused by the dilution of the catalase during cell division in the early incubation stage and the enhancement of catalase activity in the log phase. Similar enzymatic behavior was also found in *V. fischeri* ETBB10-1 biofilm. Using FEM, a simulation model was established, and the amount of the decomposed H_2_O_2_ was estimated to be 3 × 10^6^ molecules per bacterium per second. Notably, the biofilm was considered to be uniform so that the decomposition of H_2_O_2_ at the biofilm surface can be described as a constant flux state. The methodology described in this study is valuable for understanding the impact of catalase activity in symbiotic bacteria during colonization.

This finding gives an important estimate of the amount of H_2_O_2_ that is produced at the bacteria surface, which may be useful for elucidating the defense mechanism of a bacterial species or interactions with other bacterial species. However, microorganism metabolism inevitably complicates the solution system, which could reduce the accuracy of probe detection. Planktonic bacteria and some irrelevant substances may adsorb on the surface of the probe, thus decreasing the probe sensitivity, which could be particularly problematic for the long-time monitoring of the metabolic activities of biofilms.

The successful application of SECM to study bacterial aggregation is the first step toward understanding the inner world of biofilm, which may lead to new applications such as investigating microbiologically influenced corrosion (MIC). MIC can be described as an accelerated corrosion process at a metal surface caused by the activities and metabolites of microorganisms, and it is considered the primary cause of many material failures in marine, soil, and industrial environments [173]. The latest research results indicate that MIC is essentially a bioelectrochemical process [174]. At present, electrochemical methods including electrochemical impedance spectroscopy, recording polarization curves, and electrochemical noise are widely used to indirectly study the MIC mechanism. However, in these conventional general electrochemical methods, the results reveal only macroscopic variations in substrates, whereas MIC is a localized corrosion process. Furthermore, the working electrode not only serves as a support for bacteria immobilization, but also records electrochemical signals in conventional three-electrode electrochemical platforms, which results in the absence of an independent probe to record the dynamic electrode processes. Thus, conventional electrochemical methods are limited for investigating the influence of specific chemical species such as metabolites, chemical signal molecules, and electron shuttles. Given the complexity of the biofilm, advanced approaches that can closely probe or even penetrate the biofilm are needed. SECM shows a significant potential for detecting local electrochemical variation in the vicinity of the biofilm and establishing relationships between chemical species and the electrochemical corrosion process at the metal surface. When a potential is applied at the SECM tip, the oxidation or reduction of specific chemical species in the culture media or the biofilm can be observed, and the obtained current map may be useful for explaining the interaction between biofilms [175,176].

The first feasible way to investigate the MIC using SECM is by monitoring the nutriment consumption of bacteria. Under anaerobic conditions, corrosion products such as iron oxides or H_2_ serve as the energy source for the growth of hydrogen-oxidizing bacteria and iron-reducing bacteria, which in turn influence the metallic corrosion rate. For example, Vivier et al. [177] calculated the local consumption of H_2_ by Shewanella oneidensis at the surface of low-carbon steel. Under anaerobic conditions, H_2_ was consumed by S. oneidensis at a rate of $10^{-14}\;\mathrm{mol}\cdot s^{-1}$, with only corrosion products as energetic substrates. However, this experiment did not correlate the variation in H_2_ consumption with carbon steel corrosion.

Another important SECM application in MIC studies is the detection of certain enzymatic activities in biofilms. The significant influences of the enzymatic reactions on MIC have been described in previous studies [178,179]. These enzymes are believed to contribute greatly to accelerating the corrosion process by regulating the physiological activity of the biofilms. The unique characteristics of SECM make it a potential tool for performing local electrochemical measurements and investigating the enzymatic activities in the biofilms. Aside from different enzymes, certain metabolic products and pH variation near or inside the extracellular polymeric substances (EPS) may become the focus of interest in SECM-based MIC studies.

Although preliminary investigations of MIC have been conducted, several major issues remain that must be addressed regarding the SECM platforms in MIC studies. For instance, the main limitation is the uncontrollable environmental conditions in SECM. To track variations in biological and electrochemical processes, the samples are typically immersed in nutrient-rich culture media for a long time. Therefore, proper containment of the experimental platform is necessary in order to avoid contamination by foreign bacterial species from the atmospheric environment. In addition, an air supply and exhaust system can be used to provide the anaerobic and aerobic conditions. Another main limitation is the relatively low temporal resolution, since the in situ detection of MIC is a time-dependent process. Improving the high-speed SECM mode is certainly another direction for future progress in biological systems, especially for an in situ MIC study [180,181].

## 4. Conclusions and Future Perspectives

In this review, we have summarized the recent progress in SECM as a platform for studying different biological systems, particularly those associated with enzymatic activity, DNA detection and analysis, the physiological activity of living cells, bacterial behavior, and biofilm activities. Compared with electron beam-based optical imaging methods, imaging via SECM avoids the influence of diffraction, which guarantees the application of SECM on either a conducting or insulating surface. Over the last 30 years, tremendous progress has been made in the fabrication of sophisticated, miniaturized SECM probes, and in precisely controlling the probe movement. As the probe size decreases to the nanoscale, several advantages have been confirmed in biological systems, including exceptional spatial resolution and the ability to conduct electrochemistry measurements in intracellular spaces. With available resolution of approximately tens of nanometers at present, one can monitor the ion fluxes through the membrane of an individual cell or investigate different cell–cell interactions in biofilms. Furthermore, different combined analysis methods have been presented, such as SICM-SECM, AFM-SECM, and SKP-SECM. Due to their capability for dual measurement and high spatial resolution, the topographical and electrochemical mapping of different biological systems can be achieved. With the aid of these techniques, we can extend the uses of SECM to more domains, and obtain more comprehensive information on biological systems.

To reach the full potential of SECM in biological applications, future endeavors are needed to improve the capacity of SECM to solve biologically relevant issues in a more controlled cell environment and a relatively short time. The integration of photoacoustics and fluorescence with a combined SECM analytical platform seems to be a promising way to monitor the state of cells during SECM measurements. Supplementary components such as isothermal and gas barrier units can be assembled in the SECM platform and used to better control the microenvironment for cell growth during an investigation. Another pressing issue is the temporal resolution of SECM measurements. Since biological systems are always changing dynamically, temporal resolution is critical to the real-time monitoring of substrates. Notably, how to establish accurate numerical simulations of diffusion and convection processes is crucial to accelerating the scanning speed.
